# Frovatriptan and Rizatriptan Economic EVAluation: the FREEVA study

**DOI:** 10.1186/1129-2377-14-96

**Published:** 2013-12-11

**Authors:** Carlo Lisotto, Mario Guidotti, Dario Zava, Lidia Savi

**Affiliations:** 1 Department of Neurology, Pordenone Hospital, Ospedale Civile, Via Savorgnano 2, 33078 San Vito al Tagliamento (PN), Pordenone, Italy; 2 Department of Neurology, Valduce Hospital, Como, Italy; 3 Medical Department, Istituto Lusofarmaco d’Italia, Peschiera Borromeo, Milano, Italy; 4 Department of Neurosciences, University of Turin, Turin, Italy

**Keywords:** Migraine, Frovatriptan, Rizatriptan, Pharmacoeconomy, Cost-effectiveness

## Abstract

**Background:**

The present pharmacoeconomic study compared the direct and indirect costs of using frovatriptan versus rizatriptan in the acute treatment of migraine.

**Methods:**

Data on the cost-efficacy of the two triptans were derived from a recently published Italian, multicenter, randomized, double-blind, cross-over patient preference study, comparing frovatriptan versus rizatriptan. The direct costs were obtained by calculating the drug consumption, both of triptans and rescue medications. Prices of currently marketed drugs were obtained from Italian Drug Agency price list. The indirect costs were those related to absenteeism from the workplace due to migraine.

**Results:**

129 of the 148 patients with a current history of migraine randomized to the two study drugs and completing the study were analyzed. The number of attacks treated with only 1 dose of study drug was higher with frovatriptan (157 vs. 147), whereas the number of attacks treated with ≥2 doses of study medication was higher with rizatriptan (122 vs. 110 and 74 vs. 67, respectively). However, more patients treated with frovatriptan took a rescue medication (71 vs. 59). The total direct cost per attack (including study drug rescue medication) was 9.12 € for frovatriptan and 13.54 € for rizatriptan (p < 0.05 between-treatments). As for indirect costs, in the group of patients treated with frovatriptan the mean number of lost working hours was significantly (p < 0.05) lower (1.5 h) compared to the subjects who used rizatriptan (2.8 h). Based on the earned income per unit of work, indirect costs per attack resulted to be 24.55 € for frovatriptan and 45.84 € for rizatriptan. Overall, the total costs, including direct and indirect costs, were evaluated to be 33.67 € for frovatriptan and 59.38 € for rizatriptan, respectively.

**Conclusions:**

Within the limitations of this model analysis, frovatriptan was found to be significantly more cost-effective than rizatriptan. This outcome can be explained by the lower acquisition cost of frovatriptan, the need for fewer doses, and the loss of fewer working hours. This finding could drive selection of the most appropriate oral treatment for acute migraine attacks based on both individual patient’s needs and the cost-effectiveness of the available drugs.

**Trial registration:**

2006-002572-17 (EudraCT).

## Background

Migraine is a common, chronic, neurovascular disorder characterized by recurrent attacks of headache and associated symptoms. Studies conducted around the world have consistently shown that migraine affects approximately 12–15% of the general adult population. This disorder is widespread across the world, mostly affecting young and middle-aged people, and is two-to three-times more common in women than in men [1,2]. Migraine patients differ in their management needs, largely due to the variation in severity of symptoms and their impact on the sufferer. Acute medications are needed by all migraineurs for symptomatic treatment and, for the majority of patients who have infrequent attacks, are the only therapy required. The migraine-specific medications that have become known as the triptans have revolutionized the acute treatment of migraine headache during the past 20 years. Triptans are the first-choice drugs for moderate-to-severe migraine attacks in all the management guidelines published in several countries, including the USA, UK, Italy, Canada, Germany and France. Triptans are selective 5-HT_1B_ and 5-HT_1D_ receptor agonists. Seven oral triptan formulations are now available for the treatment of migraine, each with its own characteristic strengths over a range of treatment attributes. Frovatriptan is the newest addition to the triptan class: its mean half-life is 26 h, the longest in the triptan group. The molecule was selected for development based upon its distinctive pharmacologic characteristics, which suggested that it would have the clinical potential for a long duration of action [3-5], and a low likelihood of side effects [6,7] and drug interactions [3,4,8,9]. This therapeutic profile makes this triptan particularly suitable for treating patients whose migraine attacks last a long time, with an associated high risk of headache recurrence. In the new guidelines for controlled trials of drugs in migraine established by the International Headache Society (IHS), relapse (recurrence) is deemed to be a major problem with all effective migraine treatments and should be recorded as an important efficacy index [10]. Recent trials have confirmed that frovatriptan has the lowest recurrence rate, when compared to other triptans [6]. Moreover, due to its prolonged duration of action, frovatriptan provides a higher sustained pain response [11-13]. Triptans have shown to be highly effective, well tolerated and the most cost-effective migraine therapy in patients with severe symptoms and disability [14]. In head to head comparative trials the patients’ preference for one triptan or the other was not linked to pain-free rates. The drugs showed similar efficacy in the short-term, but frovatriptan seems to be unique in the triptan class, having the longest duration of action and the lowest recurrence rate [7,15,16]. The good long-term efficacy of frovatriptan supports its indication for those patients requiring a prolonged duration of action, with a sustained effect and less side effects [5-7,15]. Rizatriptan is one of the most widely used triptans for the acute treatment of migraine. A systematic review and meta-analysis of the available triptans conducted in 2001, evaluating 53 double-blind, randomized, placebo-controlled trials, showed rizatriptan to be associated with the highest 2-h pain-free rates [17]. Rizatriptan is rapidly absorbed after oral administration; the bioavailability is approximately 40-45% and Tmax is about 1–1.5 hours [18].

The costs due to migraine (episodic and chronic) have been reported in several papers. The impact of migraine is a problem of enormous proportion, both for individual subjects and society [19]. The total indirect costs were calculated to be 1.1 billion $ per year in the US and more than 3 billion € per year in Europe [20-24]. Comparisons between triptan treatments, in terms of the cost to treat a single attack, were appraised in some European Countries. Whereas in France no difference between cost-efficacy of rizatriptan and frovatriptan was noted, in other three European Countries (Italy, Germany and UK) frovatriptan was shown to be associated with a lower cost per attack, with a significantly lower intake of rescue medications in the 24 hours following the triptan first dose [25-28]. The aim of this study was to compare direct and indirect costs for frovatriptan compared with rizatriptan in the acute treatment of migraine, based on the patients’ preference for one or the other of the comparative drugs.

## Methods

The cost analysis of drugs was conducted through a structured decision tree, built up taking into account the National Healthcare System perspective. Data on the cost-effectiveness of drugs and the direct and indirect costs were derived from a clinical study by Savi et al. [15].

### Study population

The subjects eligible for participation in the study were adults ≥18 and ≤65 years of age with a current history of migraine with or without aura according to the International Classification of Headache Disorders – second edition (ICHD-II), with at least one episode per month during the last 6 months prior to entering the study. Individuals with any contraindication to triptans or any severe or disabling medical condition (such as uncontrolled hypertension and cardiac, vascular or severe liver or renal impairment) could not be enrolled. Subjects were also excluded if they had a history of alcohol, analgesic or psychotropic drug abuse, a known hypersensitivity to the study drugs, a previous inadequate response to at least two triptans, and if they were currently using ergotamine or MAO-inhibitors, or had tension-type headache on more than six days per month. Pregnant women, breastfeeding mothers, and women of childbearing age with a positive or missing pregnancy test were not eligible. The study protocol and informed consent form were reviewed and approved by the independent institutional review board of each participating institution. Written informed consent was obtained for each subject.

### Study design

**Figure 1:**
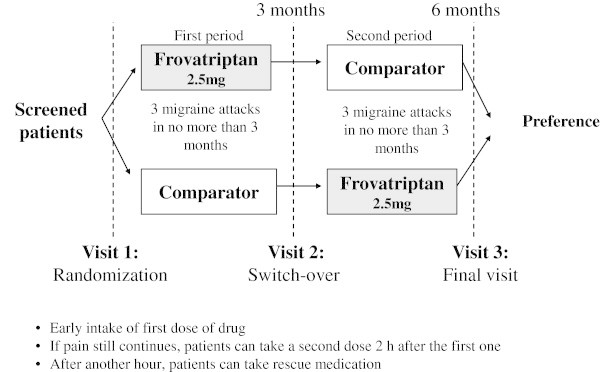
Study design.

This was a multicenter, randomized, double-blind, cross-over study, conducted in 15 centers across Italy. Each patient received the two comparative study treatments in sequence, being the sequence determined by randomization. After having treated 3 episodes of migraine in not more than 3 months with the first treatment, the patient was switched to the other treatment. After having treated 3 episodes of migraine in not more than 3 months with the second treatment, the patient was requested to indicate the preference for the first or second treatment, on a visual analogue scale (Figure 1). The primary endpoint of the trial was to evaluate the average strength of preference expressed by the patient on the visual analogue scale for the first or second treatment received. Furthermore patients completed Migraine Disability Assessment Scale (MIDAS) and Personal Preferences Questionnaire (PPQ). The details of the trial methods and all the results (primary and secondary endpoints) are reported elsewhere [15].

### Treatment

The subjects were recommended to take the first dose of study medication as early as possible after the onset of migraine attack. In case of unsatisfactory or none response after two hours, a second dose of study medication was allowed. If the relief was still insufficient, the patients could take the rescue medication (triptans and ergot derivatives excluded) one hour after the second dose. Patients who used triptans as rescue medications were excluded from the per protocol analysis and were included in the intention-to-treat analysis.

### Pharmacoeconomic data

#### Direct costs

Direct costs include all of the costs of diagnosing and treating a disease. In the case of migraine, this includes health care utilization figures such as rates of outpatient visits, hospitalization, the use of emergency department services, and costs of prescriptions. The latter were taken into account to the purpose of this survey. The analysis was performed in accordance with the published Italian guidelines [29,30]. The straightforward economic quantification of direct costs was represented by the drug consumption, both of triptans and rescue medications. The fixed or defined daily dose (DDD) is the recommended daily dose of the drug in the adult population. This dose is 2.5 mg for frovatriptan [31] and 10 mg for rizatriptan [32]. The direct costs were calculated multiplying the number of DDDs taken in the two study arms by the corresponding DDD cost. Prices of currently marketed drugs were obtained from Italian Drug Agency price list [33].

#### Indirect costs

Indirect costs include the aggregate effects of migraine on productivity at work, and in other roles. Many migraine sufferers miss work because of their headaches, and reduced productivity as a result of working during a migraine is common. Loss of productivity can be assessed measuring how much time patients have lost on workplace for their usual activities. Using a human capital approach, indirect costs can be expressed as costs incurred due to absenteeism from the work place. To determine the cost related to a lost working day, the average Italian yearly salary, i.e. 28,811.20 € [34], was divided by the mean number of working days per year, which in Italy is 220. The average cost per day thus was estimated to be € 130.96. A typical working day includes 8 productive hours.

#### Data analysis

Results are reported using proportions for categorical data, mean as central tendency parameters for continuous data and standard deviation (SD) value as dispersion parameters. Costs were reported as mean €/migraine attack. In order to compare results obtained from the two study arms, a t-test was used and p-values lower than 0.05 were kept for statistical significance. All analyses were performed using Statistical Analysis System (SAS 9.2).

## Results

### Study population

**Figure 2:**
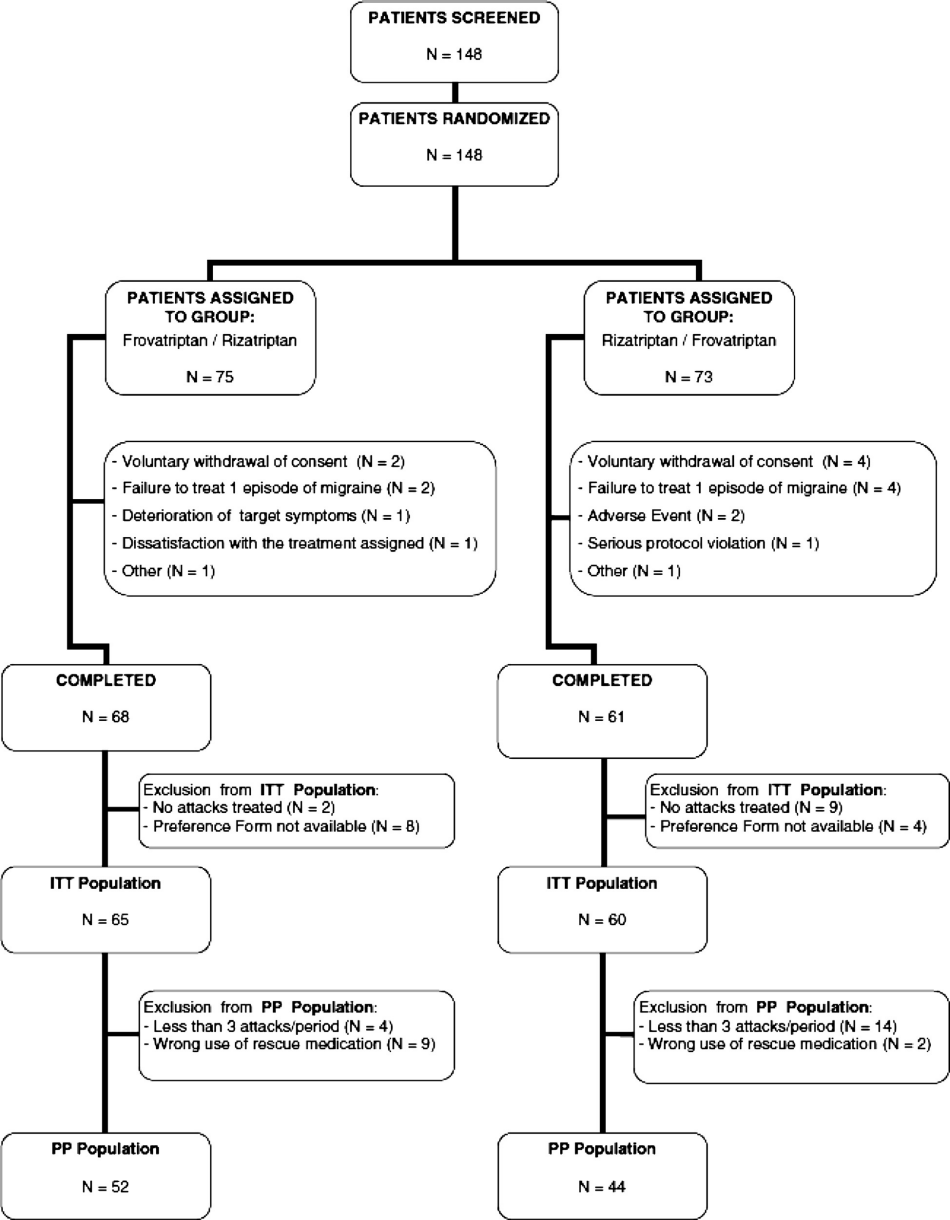
Flow diagram of participants throughout the main study.

A total of 148 patients with a current history of migraine were screened and randomized to the two study groups. A flow diagram of the patients throughout the study is reported in Figure 2. Of these subjects, 129 completed both trial periods. Nineteen patients withdrew from the study for the following reasons: dissatisfaction to assigned treatment (n = 1), withdrawal of consent (n = 6), failure to treat one episode of migraine (n = 6), occurrence of an adverse event (n = 2), protocol violation (n = 1), deterioration of target disease symptoms (n = 1), or other reasons (n = 2). Patients considered valid for the economic evaluation were the intention-to-treat population, which included 125 subjects [15].

### Main study’s results

No significant differences in terms of preference scores were found between the two study medications (3.2 ± 1.1 for rizatriptan vs. 2.9 ± 1.3 for frovatriptan). The patients who have expressed their preference for frovatriptan reported as main factors for their choice: rapidity of action (71%), tolerability (42%), reduction in pain severity (33%), complete analgesia (33%) and functional recovery (33%). The patients’ reasons for preferring rizatriptan were: rapidity of action (66%), reduction in pain severity (54%), complete analgesia (54%), tolerability (38%) and functional recovery (36%). The most relevant finding resulting from the study was the significantly lower recurrence rate within 48 h for frovatriptan (21%) vs. rizatriptan (43%); this difference was statistically significant (p < 0.001). Table 1 summarizes clinical and demographic data of the intention-to-treat population.

**Table 1:** **Demographics and clinical data of the intention**-**to**-**treat** (**ITT**) **population**[13]

	**ITT (N** = **125)**
Age (years, mean ± SD)	37 ± 9
Females (n, %)	99 (79)
Height (cm, mean ± SD)	167 ± 9
Weight (kg, mean ± SD)	64 ± 13
Age at onset of migraine (years, mean ± SD)	16 ± 7
Migraine with aura (n, %)	4 (3)
MIDAS score (mean ± SD)	22 ± 15
Migraine attack duration >2 days (n, %)	26 (21)

Data are shown as mean (±SD), or absolute (n) and relative frequency (%).

### Direct costs

Patients were treated with 1 or 2 doses of study medications per attack, either oral frovatriptan 2.5 mg or oral rizatriptan 10 mg; these formulations correspond with the DDD dosages [33].

Patients were allowed to take a rescue medication if the study treatment could not abort the migraine attack within 3 hours of its onset, one hour after the possible second dose of the study drug. During the 6-month study period, 719 migraine episodes were recorded, 357 in the frovatriptan arm and 362 in the rizatriptan arm [15]. The number of attacks and the number of medications used to treat the attacks in each arm of the cross-over study are illustrated in Table 2.

**Table 2:** Rescue medication list

	**Cross**-**over study arm**		**Total**
	**Frovatriptan**	**Rizatriptan**	
**Active compounds**	**n (%)**	**n (%)**	**n (%)**
Ibuprofen	25(23.2)	23(24)	48(23.5)
Nimesulide	21(19.4)	15(15.6)	36(17.7)
Paracetamol/codeine	13(12)	8(8.3)	21(10.3)
Ketoprofen	7(6.48)	9(9.4)	16(7.8)
Diclofenac	4(3.7)	7(7.3)	11(5.4)
Indomethacin	8(7.4)	3(3.1)	11(5.4)
Naproxen	7(6.5)	3(3.1)	10(4.9)
Ketorolac	5(4.6)	4(4.2)	9(4.4)
Paracetamol	5(4.6)	2(2.1)	7(3.4)
Frovatriptan*	1(0.9)	6(6.3)	7(2.5)
Piroxicam	3(2.8)	2(2.1)	5(2)
Metamizol	1(0.9)	3(3.1)	4(2)
Metoclopramide	1(0.9)	3(3.1)	4(1.5)
Acetylsalicylic acid	3(2.8)	0	3(1)
Eletriptan*	1(0.9)	2(2.1)	3(0.5)
Indomethacin/caffeine/prochlorperazine	0	2(2.1)	2(1)
Almotriptan*	1(0.9)	1(1)	2(3.4)
Sumatriptan*	0	2(2.1)	2(1)
Propiphenazon/butalbital/caffeine	1(0.9)	0	1(0.5)
Rizatriptan*	0	1(1)	1(1.5)
Betamethasone	1(0.9)	0	1(0.5)
**Total**	108	96	204

*Patients who used triptans as rescue medications were excluded from the per protocol analysis and were included in intention-to-treat analysis.

Taking into account only the triptan use, the cost for treating migraines was 3,170 € for frovatriptan (357 attacks) and 4,782 for rizatriptan (362 attacks).

One hundred and thirty of the 719 triptan-treated migraine attacks required the use of rescue medication (further triptans and ergot derivatives were excluded by protocol). Rescue medication was taken by 19.9% of subjects in frovatriptan arm and by 16.3% of patients in rizatriptan arm. A list of all active compounds used as rescue medication in either arm is shown in Table 2. The most commonly used rescue medications were non-steroidal anti-inflammatory drugs, in particular:

• Salicylates, 2.8% in frovatriptan arm and 0% in rizatriptan arm;

• Propionic acid derivatives, 36.2% in frovatriptan arm and 36.5% in rizatriptan arm

• Acetic acid derivatives, 44.8% in frovatriptan arm and 11.5% in rizatriptan arm

• Enolic acid (Oxicam) derivatives, 2.8% in frovatriptan arm and 2.1% in rizatriptan arm;

• Selective COX inhibitors, 12% in frovatriptan arm and 5.2% in rizatriptan arm

• Sulphonanilides, 19.4% in frovatriptan arm and 15.6 in rizatriptan arm;

• Others, 13.8% in frovatriptan arm and 13.5% in rizatriptan arm

Other rescue medications used were: antiemetics (1.0% in frovatriptan arm and 3.6% in rizatriptan arm) and corticosteroids (1.0% frovatriptan arm; 0% rizatriptan arm). The total cost of rescue medications was 85.7 € for the patients who used frovatriptan and 119.46 € for the subjects treated with rizatriptan. The total direct costs, consequently, were expressed adding the study drug treatment and the rescue medication costs together.

The total cost per attack was calculated to be 9.12 € for frovatriptan (0.24 € for rescue medication) and 13.54 € for rizatriptan (0.33 € for rescue medicine).

### Indirect cost

Treatment with frovatriptan also had a positive impact on the magnitude of working hours lost due to migraine attacks. In the group of patients treated with frovatriptan the mean number of lost working hours was significantly (p < 0.05) lower (1.5 h) compared to the subjects who used rizatriptan (2.8 h).

Evaluating the average loss of productivity [15] and the earned income per unit of work [34], applied to the number of migraine attacks observed during the study, the costs due to lost working days in the two treatment groups were estimated to amount to 8,766.13 € for frovatriptan (357 attacks) and 16,592.63 € for rizatriptan (362 attacks). Indirect costs per attack were 24.55 € for frovatriptan and 45.84 € for rizatriptan.

### Total costs

**Figure 3:**
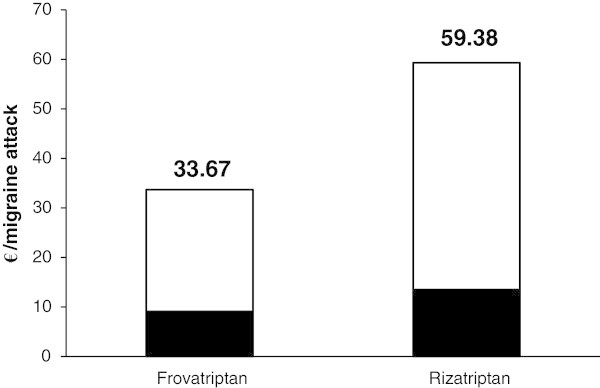
**Direct (full bars) and indirect (open bars) costs per migraine attack.** Total costs are reported on top of each bar.

Overall, total costs, including direct and indirect costs, were evaluated to be 33.67 € for frovatriptan and 59.38 € for rizatriptan, respectively, as shown in Figure 3.

## Discussion

Triptans ushered in a new era in acute migraine therapy, with their ability to provide rapid relief of headache and associated symptoms. With the introduction of triptans, migraine therapy has made a quantum leap forward [11]. Triptans are currently the first line drugs for the acute treatment of migraine [14] as they have revolutionized the management of this disorder. Since the introduction of the first triptan, sumatriptan, launched over 20 years ago, six other triptans with distinctive pharmacokinetic properties have been developed [11]. Although all triptans have the same mechanism of action and simple and consistent pharmacokinetic features, there are specific differences among individual agents that may account for their different clinical attributes. For instance, in the treatment of perimenstrual migraines, which are known to be particularly severe and disabling, the different triptans showed different profiles of efficacy, either given for acute attacks or for short-term prophylaxis [35]. In the patient preference head to head comparative trials, the drugs showed similar efficacy in the short-term, in particular at 2 hours, but frovatriptan provided a more sustained response, with a lower recurrence rate, likely related to its much longer elimination half-life [6]. The pooled systematic analysis of the three preference trials conducted in Italy suggests that frovatriptan has a similar antimigraine efficacy to other widely employed triptans (rizatriptan, zolmitriptan and almotriptan). This is true of both the immediate and the sustained pain-relieving effects. Frovatriptan has also been shown to be better tolerated than the other studied triptans [6].

In this pharmacoeconomic study frovatriptan treatment has shown to be less expensive than rizatriptan, despite the slightly higher number of rescue medication doses required. This outcome can be explained by a combination of the lower number of doses needed, on average, to treat a migraine attack, and the lower number of hours lost from the workplace when using frovatriptan.

There are two important limitations of this study: first of all, that this is not a pharmacoeconomic study, but a sub-analysis not provided in statistical plan and secondly this analysis was based on the human capital approach. This approach suggests that health care interventions are a kind of investment in an individual’s human capital (similar to education). According to this method, evaluation of productivity losses is based on labor costs. All future productivity losses (up to retirement age) are considered in the human capital approach, but obviously it represent a selection of costs included in the model, so some other costs aren’t included.

## Conclusions

On the basis of published data [23,29] and with the model analysis based on the human capital approach, results suggest the economical advantage of frovatriptan 2.5 mg among the oral triptans approved for the treatment of migraine in Italy. In this study, in particular, frovatriptan was found to be significantly more cost-effective than rizatriptan. This outcome can be explained by the lower acquisition cost of frovatriptan, the need for fewer doses, and the loss of fewer working hours. This finding could drive selection of the most appropriate oral treatment for acute migraine attacks, based on both individual patient’s needs and the cost-effectiveness of the available drugs.

## Competing interests

All authors have occasionally served as scientific consultants for manufacturers of frovatriptan or rizatriptan. Dario Zava is an employee from the manufacturer of frovatriptan.

## Authors’ contributions

CL, LS, MG and DZ participated in data collection, management and analysis. CL analyzed the final data. CL and DZ wrote the manuscript. All authors read and approved the final manuscript.

## References

[B1] Lipton RB Bigal ME Migraine: epidemiology, impact, and risk factors for progression Headache 2005 14 suppl.1 S3 S13 15833088 10.1111/j.1526-4610.2005.4501001.x

[B2] Rasmussen BK Jensen R Schroll M Olesen J Epidemiology of headache in a general population - a prevalence study J Clin Epidemiol 1991 14 11 1147 1157 10.1016/0895-4356(91)90147-2 1941010

[B3] Goldstein J Frovatriptan: a review Expert Opin Pharmacother 2003 14 1 83 93 10.1517/14656566.4.1.83 12517245

[B4] Markus F Mikko K Frovatriptan review Expert Opin Pharmacother 2007 14 17 3029 3033 10.1517/14656566.8.17.3029 18001261

[B5] Allais G Tullo V Benedetto C Zava D Omboni S Bussone G Efficacy of frovatriptan in the acute treatment of menstrually related migraine: analysis of a double-blind, randomized, multicenter, Italian, comparative study versus zolmitriptan Neurol Sci 2011 14 suppl.1 S99 S104 21533723 10.1007/s10072-011-0547-y PMC3084939

[B6] Cortelli P Allais G Tullo V Benedetto C Zava D Omboni S Bussone G Frovatriptan versus other triptans in the acute treatment of migraine: pooled analysis of three double-blind, randomized, cross-over, multicenter, Italian studies Neurol Sci 2011 14 suppl.1 S95 S98 21533722 10.1007/s10072-011-0551-2

[B7] Bartolini M Giamberardino MA Lisotto C Martelletti P Moscato D Panascia B Savi L Pini LA Sances G Santoro P Zanchin G Omboni S Ferrari MD Brighina F Fierro B A double-blind, randomized, multicenter, Italian study of frovatriptan versus almotriptan for the acute treatment of migraine J Headache Pain 2011 14 3 361 368 10.1007/s10194-011-0325-5 21437714 PMC3094646

[B8] Rapoport A Ryan R Goldstein J Keywood C Dose range-finding studies with frovatriptan in the acute treatment of migraine Headache 2002 14 suppl.2 S74 S83 12028323 10.1046/j.1526-4610.42.s2.5.x

[B9] Buchan P Keywood C Wade A Ward C Clinical pharmacokinetics of frovatriptan Headache 2002 14 suppl.2 S54 S62 12028321 10.1046/j.1526-4610.42.s2.3.x

[B10] Tfelt-Hansen P Pascual J Ramadan N Dahlöf C D'Amico D Diener HC Hansen JM Lanteri-Minet M Loder E McCrory D Plancade S Schwedt T Subcommittee IHSCT Guidelines for controlled trials of drugs in migraine: third edition. A guide for investigators Cephalalgia 2012 14 1 6 38 22384463 10.1177/0333102411417901

[B11] Loder E Triptan therapy in migraine N Engl J Med 2010 14 1 63 70 10.1056/NEJMct0910887 20592298

[B12] Tepper SJ Rapoport AM Sheftell FD Mechanism of action of the 5-HT_1B/1D_ receptor agonists Arch Neurol 2002 14 7 1084 1088 10.1001/archneur.59.7.1084 12117355

[B13] Balbisi EA Frovatriptan succinate, a 5-HT_1B/1D_ receptor agonist for migraine Int J Clin Pract 2004 14 7 695 705 10.1111/j.1368-5031.2004.00218.x 15311727

[B14] Lantėri-Minet M Clinical use of triptans in the management of migraine CNS Drugs 2006 14 Special no 1 12 23 16841523

[B15] Savi L Omboni S Lisotto C Zanchin G Ferrari MD Zava D Pinessi L A double-blind, randomized, multicenter, Italian study of frovatriptan versus rizatriptan for the acute treatment of migraine J Headache Pain 2011 14 2 219 226 10.1007/s10194-010-0243-y 20686810 PMC3075392

[B16] Géraud G Keywood C Senard JM Migraine headache recurrence: relationship to clinical, pharmacological, and pharmacokinetic properties of triptans Headache 2003 14 4 376 388 10.1046/j.1526-4610.2003.03073.x 12656709

[B17] Ferrari MD Goadsby PJ Roon KI Lipton RB Triptans (serotonin, 5-HT_1B/1D_ agonists) in migraine: detailed results and methods of a meta-analysis of 53 trials Cephalalgia 2002 14 8 633 658 10.1046/j.1468-2982.2002.00404.x 12383060

[B18] Gijsman H Kramer MS Sargent J Tuchman M Matzura-Wolfe D Polis A Teall J Block G Ferrari MD Double-blind, placebo-controlled, dose-finding study of rizatriptan (MK-462) in the acute treatment of migraine Cephalagia 1997 14 6 647 651 10.1046/j.1468-2982.1997.1706647.x 9350384

[B19] Mennini FS Gitto L Martelletti P Improving care through health economics analyses: cost of illness and headache J Headache Pain 2008 14 4 199 206 10.1007/s10194-008-0051-9 18604472 PMC3451939

[B20] Guidotti M Ravasio R Clinical and economic comparison of frovatriptan versus other oral triptans in the treatment of acute migraine in the real-world setting Clin Drug Investig 2009 14 11 693 702 10.2165/11315330-000000000-00000 19813772

[B21] Pradalier A Auray JP El Hasnaoui A Alzahouri K Dartigues JF Duru G Henry P Lantéri-Minet M Lucas C Chazot G Gaudin AF Economic impact of migraine and other episodic headaches in France: data from the GRIM2000 study Pharmacoeconomics 2004 14 15 985 999 10.2165/00019053-200422150-00003 15449963

[B22] Pesa J Lage MJ The medical costs of migraine and comorbid anxiety and depression Headache 2004 14 6 562 570 10.1111/j.1526-4610.2004.446004.x 15186300

[B23] Edmeads J Mackell JA The economic impact of migraine: an analysis of direct and indirect costs Headache 2002 14 6 501 509 10.1046/j.1526-4610.2002.04262.x 12167138

[B24] Hawkins K Wang S Rupnow MF Indirect cost burden of migraine in the United States J Occup Environ Med 2007 14 4 368 374 10.1097/JOM.0b013e31803b9510 17426520

[B25] Wallasch TM Frovatriptan in the practice of office-based neurologists/pain therapists: results of postmarketing surveillance study ALADIN Adv Ther 2010 14 1 56 62 10.1007/s12325-010-0001-1 20140543

[B26] Pascual J Fité B López-Gil A Comparison of triptan tablet consumption per attack: a prospective study of migraineurs in Spain Headache 2002 14 2 93 98 10.1046/j.1526-4610.2002.02024.x 12005301

[B27] Pfaffenrath V Spierungs EH Akutbehandlung von Migräneattacken. Frovatriptan 2.5 mg als effektive und ökonomische Alternative Nervenheilkunde 2004 14 9 545 548

[B28] Leira R, Dualde E, del Barrio H, Machuca M, López-Gil A; Spanish Group for the study of triptan consumption in community pharmacies Almotriptan versus rizatriptan in patients with migraine in Spain Headache 2003 14 7 734 741 10.1046/j.1526-4610.2003.03131.x 12890128

[B29] Garattini L Grilli R Scopelliti D Mantovani L A proposal for Italian Guidelines in pharmacoeconomics. The Mario Negri Institute centre for health economics Pharmacoeconomics 1995 14 1 1 6 10.2165/00019053-199507010-00001 10155289

[B30] Capri S Ceci A Terranova L Merlo F Mantovani L Guidelines for economic evaluations in Italy: recommendations from the Italian Group of Pharmacoeconomic Studies Drug Inf J 2001 14 189 201

[B31] Selective serotonin (5HT1) agonists: frovatriptan 2013 Available at: http://www.whocc.no/atc_ddd_index/?code=N02CC07&showdescription=yes

[B32] Selective serotonin (5HT1) agonists: rizatriptan 2013 Available at: http://www.whocc.no/atc_ddd_index/?code=N02CC04

[B33] Consultazione Farmaci 2013 Available at: http://www.agenziafarmaco.gov.it/

[B34] Istituto Nazionale di Statistica 2013 Available at: http://www.istat.it/

[B35] Casolla B Lionetto L Candela S D’Alonzo L Negro A Simmaco M Martelletti P Treatment of perimenstrual migraine with triptans: an update Curr Pain Headache Rep 2012 14 5 445 451 10.1007/s11916-012-0280-0 22644903

